# Difference in anisotropic etching characteristics of alkaline and copper based acid solutions for single-crystalline Si

**DOI:** 10.1038/s41598-018-21877-x

**Published:** 2018-02-21

**Authors:** Wei Chen, Yaoping Liu, Lixia Yang, Juntao Wu, Quansheng Chen, Yan Zhao, Yan Wang, Xiaolong Du

**Affiliations:** 1 Key Laboratory for Renewable Energy, Beijing Key Laboratory for New Energy Materials and Devices, National Laboratory for Condensed Matter Physics, Institute of Physics, Chinese Academy of Sciences, Beijing, 100190 China; 2 School of Physical Sciences, University of Chinese Academy of Sciences, Beijing, 100049 China

## Abstract

The so called inverted pyramid arrays, outperforming conventional upright pyramid textures, have been successfully achieved by one-step Cu assisted chemical etching (CACE) for light reflection minimization in silicon solar cells. Due to the lower reduction potential of Cu^2+^/Cu and different electronic properties of different Si planes, the etching of Si substrate shows orientation-dependent. Different from the upright pyramid obtained by alkaline solutions, the formation of inverted pyramid results from the coexistence of anisotropic etching and localized etching process. The obtained structure is bounded by Si {111} planes which have the lowest etching rate, no matter what orientation of Si substrate is. The Si etching rate and (100)/(111) etching ratio are quantitatively analyzed. The different behaviors of anisotropic etching of Si by alkaline and Cu based acid etchant have been systematically investigated.

## Introduction

Silicon (Si) surface texturing is an indispensable step for the fabrication of Si solar cells due to the high reflection of planar Si wafers. Thus, surface texture and antireflection coating such as SiN_x_ are necessary to reduce the surface reflectance of solar cells. Currently, industrialized techniques for texturing Si wafers are generally based on alkaline solutions for single-crystalline Si (c-Si) by the anisotropic etching^1^ or acid solutions for multi-crystalline Si (mc-Si) by the isotropic etching^2^. Usually, upright random sized pyramidal structures^3,4^ for c-Si and ‘worm like’ structures^5^ for mc-Si will be obtained, respectively. More specifically, the texturization of c-Si is usually based on alkali/isopropyl alcohol (IPA) mixture required processing at high temperatures (75 °C–85 °C) for about 20 minutes^3,6,7^, which had already been commercialized on (100) oriented Si wafers decades ago. It is well-known that each atom of Si {100} surface has two dangling bonds and two back bonds in contrast to one dangling and three back ones for each atom of {111} surface. Therefore, the activation energy to remove an atom from {100} surface is smaller than that from {111} surface because it only needs breaking two back bonds rather than three ones in the case of {111} surface. Thus, in alkaline etchant, the etching rate is faster along the directions of [100] than that along other directions due to the lower activation energy of the atoms on {100} surface^8^. Thereby, the etching mainly occurs along the [100] directions and stops at the {111} planes, leading to the formation of upright pyramidal structures.

The inverted pyramid texture, outperformed the pyramid texture because of their superior light-trapping and structure characteristics, were typically fabricated by a combination of alkali wet chemical etching and photolithography^9–12^. Due to the complexity and high cost of the photolithographic based patterning processes, such technique is not compatible with industrial solar cell manufacture. Recently, Lu *et al*. reported Cu assisted chemical etching (CACE) to prepare nanopore-type inverted pyramidal antireflective layers which took hours etching time^13^. Very recently, Y. Wang *et al*. discovered a new technical solution to fabricate micrometer sized inverted pyramidal structure by maskless Cu-nanoparticles assisted chemical etching in Cu(NO_3_)_2_/HF/H_2_O_2_/H_2_O solutions^14–16^, which had lower reflectivity and higher light absorption, along with a shorter etching time and a lower etching temperature during the texturing process compared with the industrial pyramidal structure texturization. Commonly, both upright pyramids and inverted pyramids are the consequence of anisotropic etching of Si (100) substrate, which are bounded by {111} crystallographic planes due to the slowest etching rate of {111} planes. The aim of this paper is to explain why alkaline solutions form upright pyramid while Cu(NO_3_)_2_/HF/H_2_O_2_ mixtures form inverted pyramid, though both methods are anisotropic etching.

In this paper, more detailed analysis will be proposed for the anisotropic etching of Si based on a HF/H_2_O_2_ process with the assistance of Cu nanoparticles by taking advantage of the anisotropic electrochemical behavior of a Si crystal. The etching mechanism of different oriented Si surfaces is systematically studied. The Si etching rate and (100)/(111) etching ratio are investigated as well. Additionally, surface morphologies and reflectivity after etching are characterized.

## Results and Discussion

**Figure 1:**
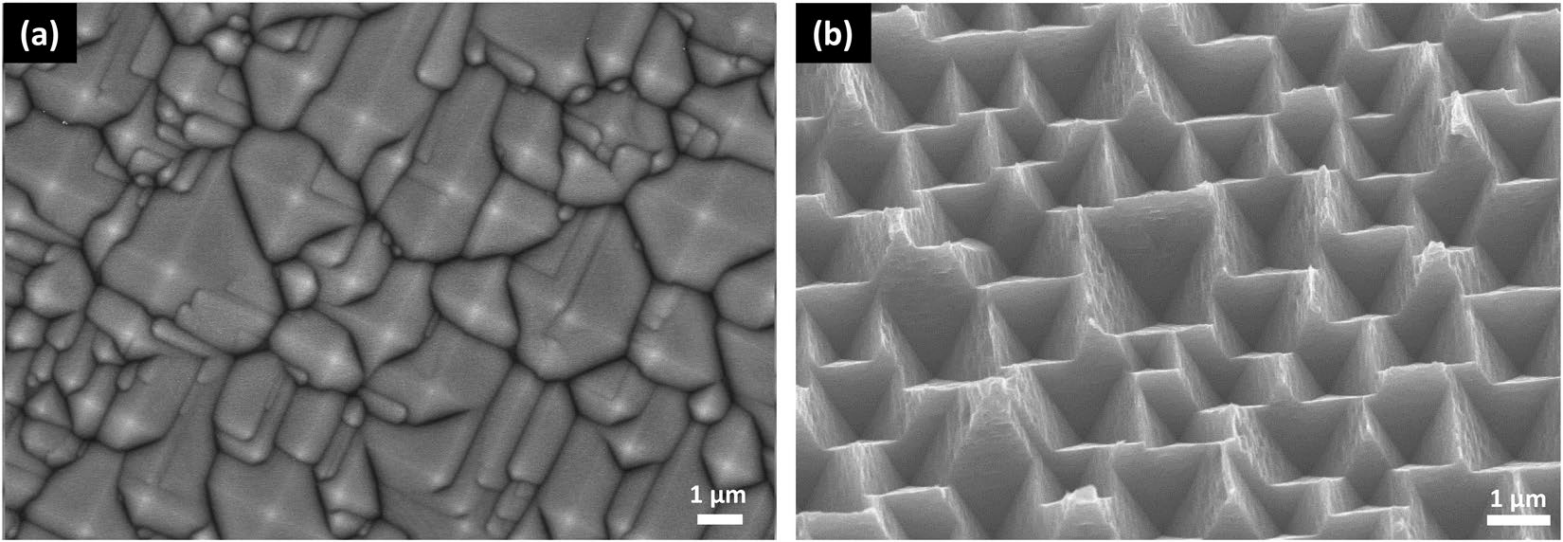
SEM images of the etched Si (100) wafers using: (**a**) alkali etching, (**b**) Cu based acid etching.

**Figure 2:**
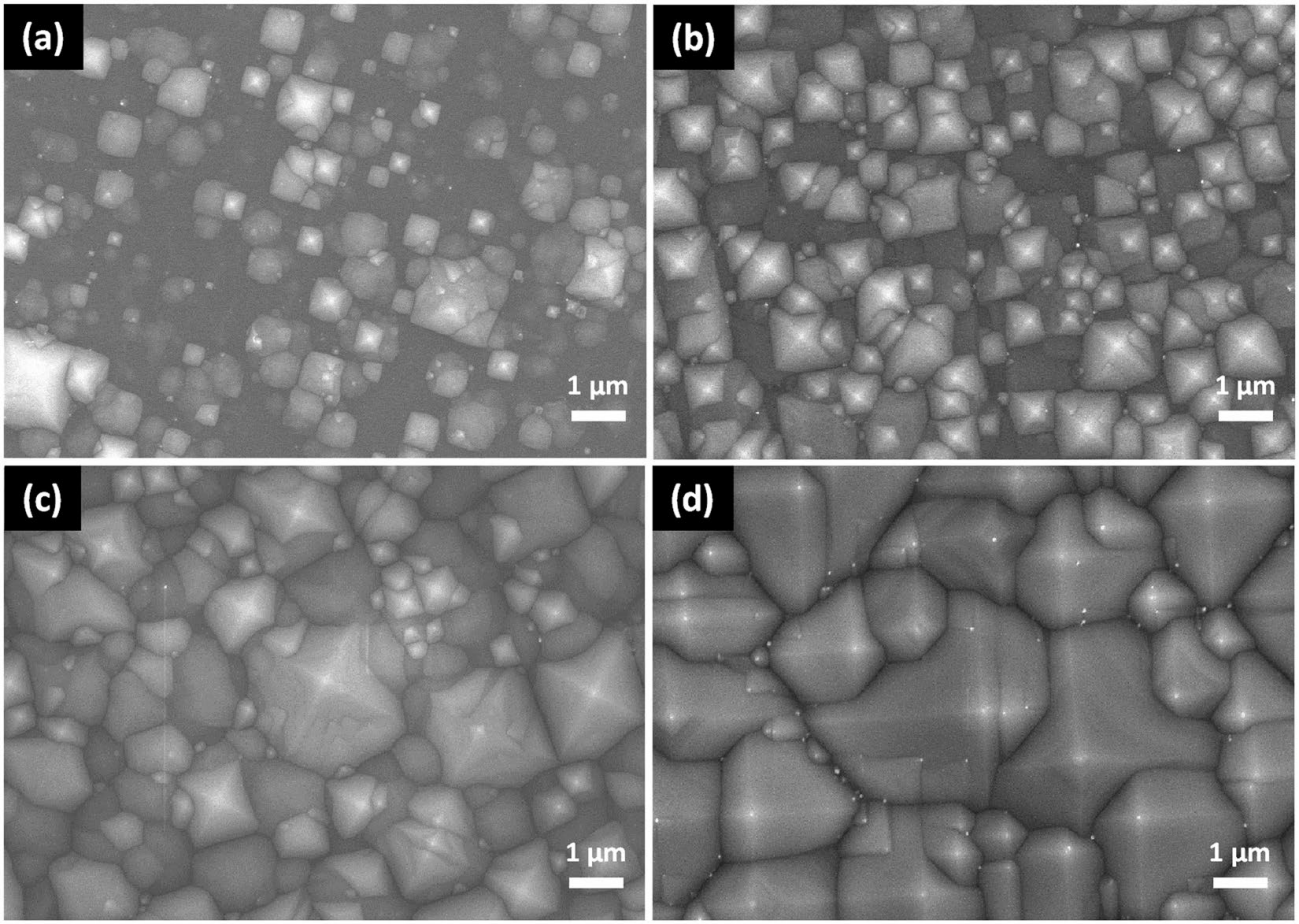
SEM images of c-Si (100) wafers etched in the 2 wt% KOH and 10 vol% IPA at 80 °C for different time: (**a**) 5 min, (**b**) 10 min, (**c**) 15 min, (**d**) 25 min.

Figure 1 shows the surface morphology of (100) Si etched by alkaline solutions for twenty-five minutes at 80 °C (Fig. 1(a)) and etched by 5 mM Cu(NO_3_)_2_, 4.6 M HF and 0.55 M H_2_O_2_ mixtures for fifteen minutes at 50 °C followed by removing of Cu nanoparticles (Fig. 1(b)). Microsized upright pyramidal structures can be observed in Fig. 1(a) while inverted pyramidal structures in Fig. 1(b). The etching rate of single crystal Si in an anisotropic etchant varies with the crystallographic orientation of the substrate, which generally decreases in the order of (100) ≈ (110) > (111)^17,18^. The alkali etching process usually includes an oxidation step and a reduction step based on an electrochemical model. During oxidation step, four hydroxide ions react with one surface atom of Si, leading to the injection of four electrons into the conduction band of Si. The reaction is accompanied by the breaking of the back bonds, which requires thermal excitation of the electrons from their surface states into the conduction band. During reduction step, the injected electrons react with water molecules to form new hydroxide. The anisotropic behavior is due to small differences of the energy levels of the back bond surface states as a function of the crystal orientation^8^. In alkaline etching process, the entire surface of Si wafers are covered by alkaline solutions and the etching is conducted on the whole Si surface. As shown in Fig. 2(a), five minutes etching in alkaline solutions leading to point structures popping on the whole surface. The point structures are formed at the kinks, steps, and other defects firstly. As the etching time increased to 10 minutes, the point structures turn into small pyramids. This process is called pyramid nucleation. When the etching time reaches 15 minutes, the whole surface is covered by pyramids, while the size of the pyramid is not uniform. As the etching time extended to 25 minutes, the pyramids further expand and merge, resulting in more uniformity of the pyramid size. The formation mechanism of upright pyramids is that the {100} crystallographic planes have lower atomic lattice packing density and more available dangling bonds than those on the {111} planes, leading to a faster etching rate along the directions of [100] rather than [111], i.e. etching mainly occurs along the [100] directions and stops at the {111} planes. Thus, the anisotropic etching behavior and the whole surface etching process lead to the formation of upright pyramid texture. Different from the etching by alkaline solutions, inverted pyramid structures were obtained on Si (100) substrate in CuNO_3_/HF/H_2_O_2_ solutions. The related electrochemical reactions can be described as two half-cell reactions analogous with the well-known metal-assisted chemical etching method for fabricating various Si nanostructures^19–23^. The deposition of Cu nanoparticles, however, demonstrates orientation-dependence on Si surface, due to the lower reduction potential of Cu^2+^/Cu (E_Cu2+/Cu_ = 0.34 V) comparing to Ag^+^/Ag (E_Ag+/Ag_ = 0.8 V) and different electronic properties of (100) and (111) planes in c-Si. More Cu nanoparticles nucleate on Si (100) plane because of the higher probability of Cu^2+^ ions to capture electrons. Si underneath the Cu nanoparticles are preferentially oxidized and then etched by HF, leading to a faster etching along Si [100] directions^11,14,15^. Thus, both the lower reduction potential of Cu^2+^/Cu and different electronic properties of (100) and (111) planes in c-Si contribute to the anisotropic etching of CACE.

**Figure 3:**
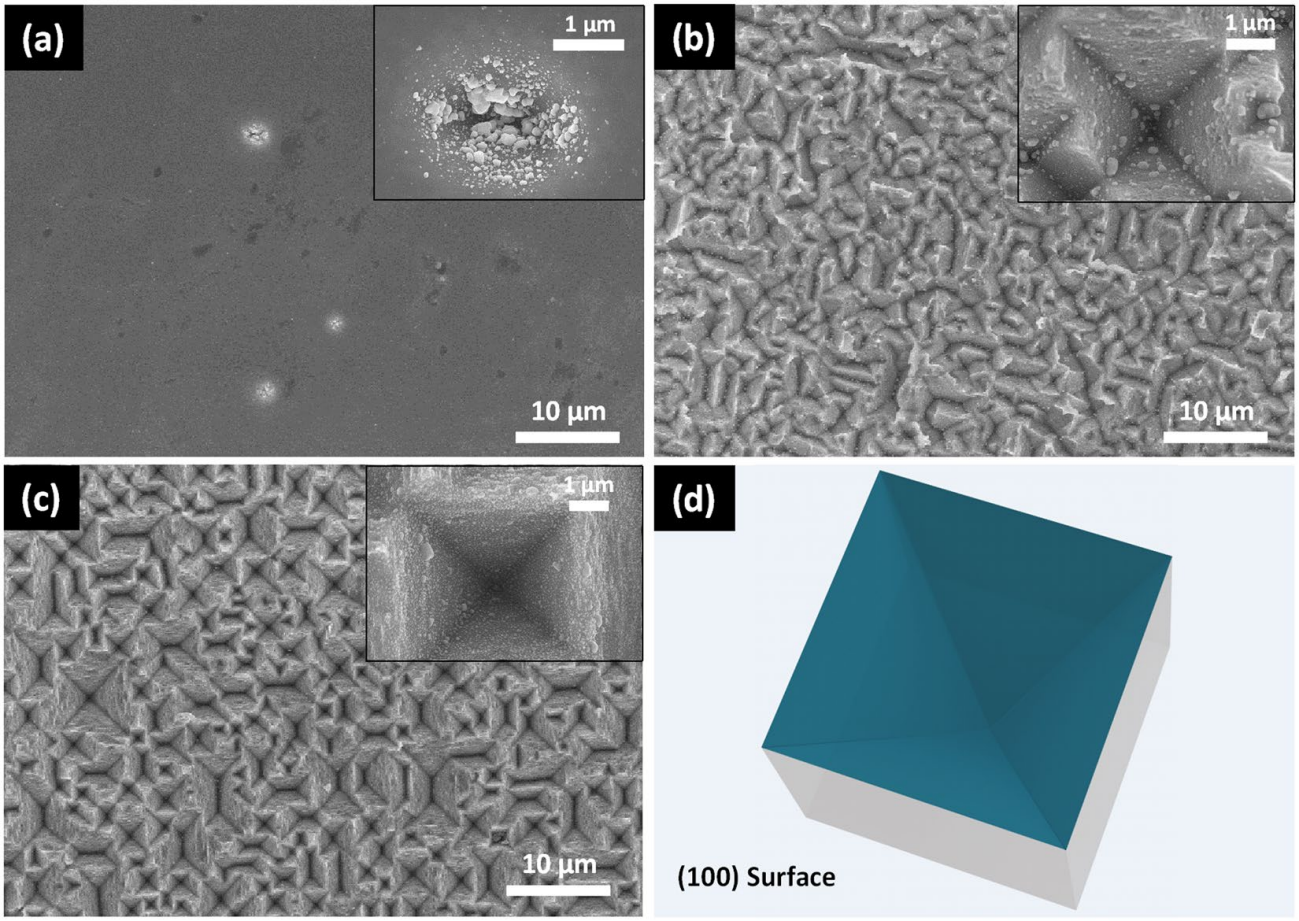
SEM images of c-Si (100) wafers etched in the 5 mM Cu(NO_3_)_2_, 4.6 M HF and 0.55 M H_2_O_2_ mixtures at 50 °C for different time: (**a**) 1 min, (**b**) 5 min, (**c**) 15 min. The insets show the corresponding amplification SEM images. (**d**) simulated schematic of Si (100) etched structure.

Figure 3 shows SEM images of c-Si (100) wafers etched in Cu based acid solutions for different time and a schematic of the simulated inverted pyramid. Generally, metal-assisted chemical etching method is accepted as a localized electrochemical process in which the oxidation and dissolution of Si only occur under the metal particles. Unlike the point structures obtained in alkaline etchant starting with the entire Si surface, the etching of Si only happened under metal particles by CACE. Small pits are formed in the early time because of the sinking of metal particles. As can be observed from Fig. 3(a), Cu nanoparticles were anisotropically deposited on Si (100) surface which leading to the formation of inverted pyramid. Obviously, only several pits were formed on the polished c-Si (100) surface. For metal assisted chemical etching, the defective sites of the Si surface function as the starting points^24,25^. Therefore, less inverted pyramids were obtained in comparison with those on the surfaces of raw Si wafers used in industry due to the less defects on polished Si surface at the early time. With the prolongation of reaction time, the region without defects could also be etched, and the size of inverted pyramid became larger, as shown in Fig. 3(b), though there are still some unetched regions. With the etching time increasing to 15 minutes, inverted pyramid became deeper and more standard across the whole surface (Fig. 3(c)). Similar geometry of cavities had been obtained by alkaline solution with opening masks previously, employed more complex techniques involving lithography, laser processes, etc^9,12,26,27^. The opening masks and the laser induced pits were used to localize the alkali etching inside the masks or pits, just like the localized electrochemical anisotropic etching of CACE. However, as the alkali etching time increased, the regular inverted pyramidal structure collapsed and tiny upright pyramids began to appear on the surface. Because the laser induced holes became shallow and the opening masks were destroyed, leading to the etching process was no longer localized inside the holes or masks. Hence, the formation of inverted pyramids not only need the anisotropic etching, but also the localized etching process.

**Figure 4:**
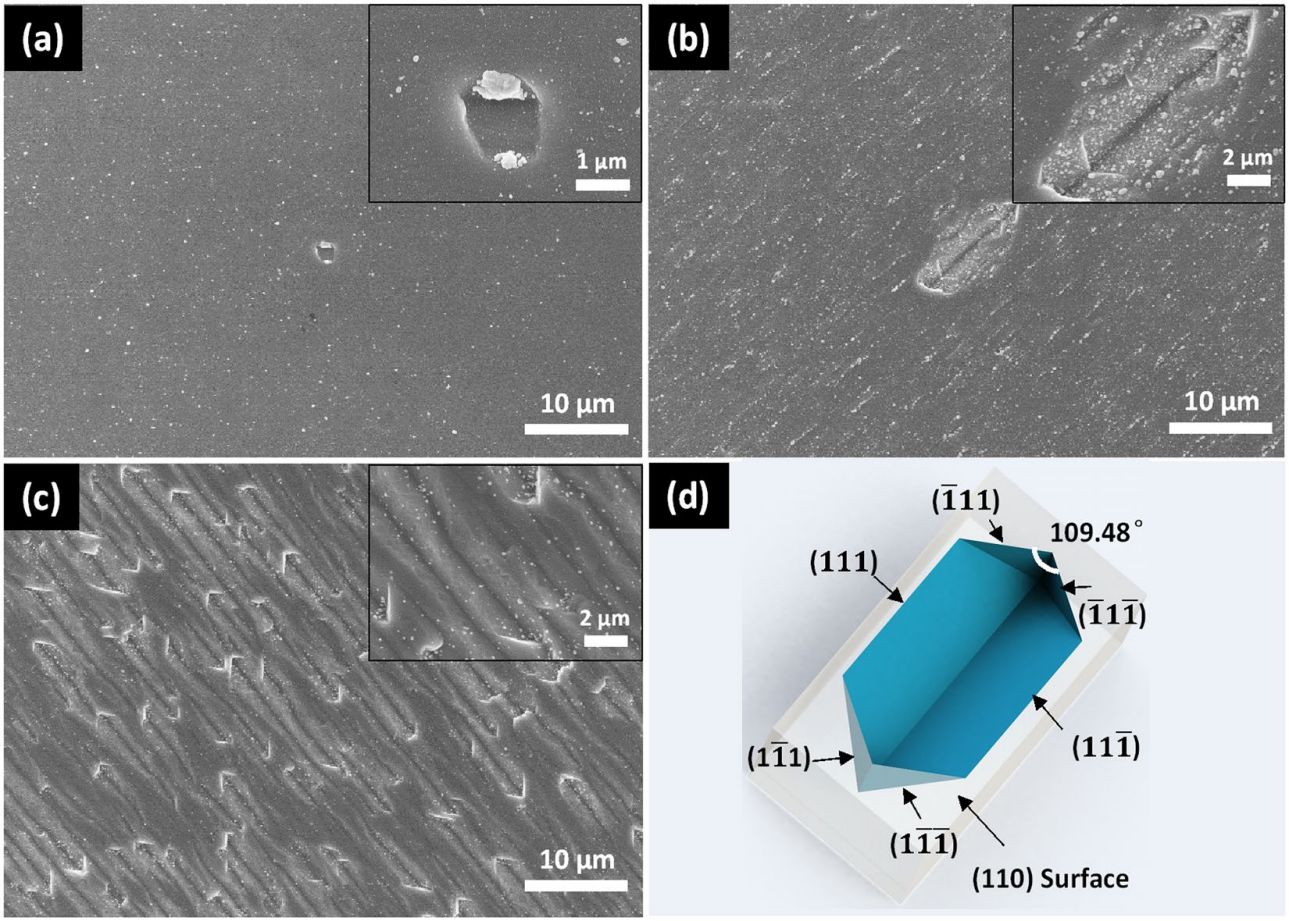
SEM images of c-Si (110) wafers etched in copper based acid solutions for different time: (**a**) 1 min, (**b**) 5 min, (**c**) 15 min. (**d**) simulated schematic of Si (110) etched structure.

In order to further verify the mechanism of CACE, the commonly used (110) and (111) oriented wafers are etched in the Cu based acid solutions. Figure 4 shows SEM images of c-Si (110) wafers fabricated by Cu based acid solutions for different time and the simulated schematic of Si (110) etched structure. The method of simulated schematic is using (110) plane to incise the {111} crystallographic planes. A similar phenomenon was observed on (110) oriented Si that the etched structure is terminated with Si {111} planes due to the slowest etching rate of {111} crystallographic planes. However, the geometries of the etched structure are different due to the fact that {111} planes which form the sidewalls of the cavities are not equivalent. Rhombus like structure was obtained on Si (110) wafers, as shown in Fig. 4. The ($\bar{1}11$) and ($\bar{1}\,$1 $\bar{1}$) planes are perpendicular to the (110) surface and intersect each other at the surface at an angle of 109.48°. The (111) and (11 $\bar{1}$) planes intersect the (110) surface at an angle of 35.26°, and intersect each other inside the cavity at 109.48°, as depict in Fig. 4(d). Also the deposition of Cu nanoparticles shows crystallographic plane orientation as shown in Fig. 4(a).

**Figure 5:**
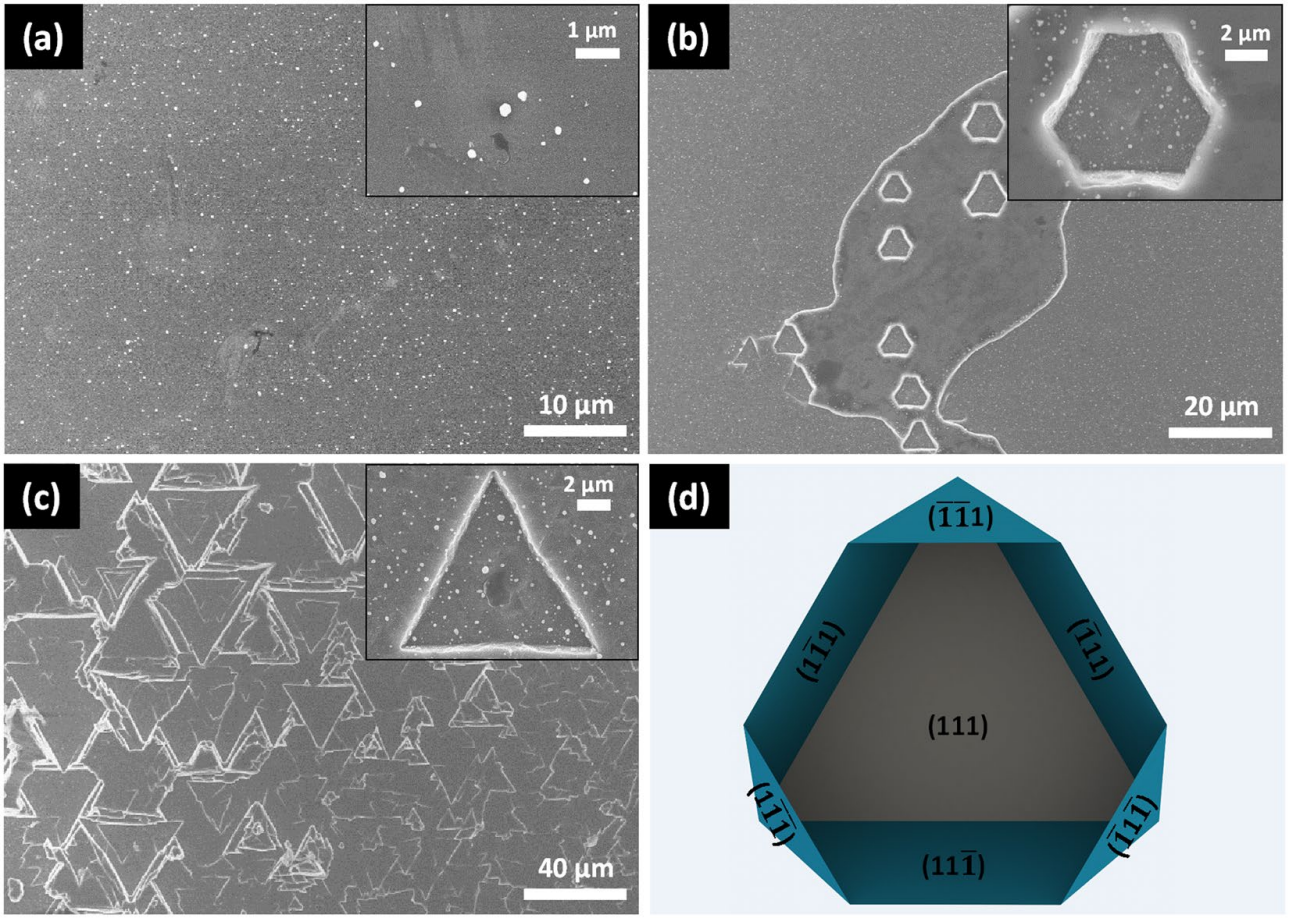
SEM images of etched c-Si (111) wafers by Cu assisted chemical etching for different time: (**a**) 1 min, (**b**) 5 min, (**c**) 15 min. (**d**) simulated schematic of Si (111) etched structure.

The (111) oriented Si is rarely used since it etches slowly in the alkaline solutions. In contrast, it is much easier to be etched by CACE. Figure 5 shows SEM images of Si (111) wafers etched in the 5 mM Cu(NO_3_)_2_, 4.6 M HF and 0.55 M H_2_O_2_ mixtures at 50 °C for different time and simulated schematic of Si (111) etched structure. The method of simulated schematic is also using (111) plane to incise the {111} crystallographic planes. Due to a lower surface dangling bond density, the Si (111) planes had less “available” electrons than Si (100) planes, Cu nanoparticles just distributed dispersedly on Si (111) surface^13–15^. The etching started at the defect sites, and the geometries of the etched structure is also bounded by Si {111} crystallographic planes. The < 111 > axis of a cubic system is an axis of hexagonal symmetry. Thus, hexagonal and triangular structure are obtained. For the hexagonal structure, as depict in Fig. 5(b,d), three {111} planes: ($1\bar{1}1$), ($\bar{1}11$) and ($11\bar{1}$) are 109.5° to the (111) surface plane, and their intersecting lines are 60° to each other. Another three {111} planes: ($\bar{1}\bar{1}1$), ($\bar{1}1\bar{1}$) and ($1\bar{1}\bar{1}$) are 70.5° to the (111) surface plane, and also the intersecting lines are 60° to each other. The six {111} planes form the walls of the hexagonally shaped cavity, whereby three {111} planes form declining facets and another three {111} planes form inclining facets with respect to the centre of the cavity^28,29^. As the etching time extended to 15 minutes, the hexagonal structure turns into equilateral triangular structure due to the continue etching of another three {111} planes. As described above, the structure obtained by the Cu-assisted chemical etching method used in this paper is bounded by Si {111} crystallographic planes whatever orientation of the Si substrate is. Besides, the etched structure can be predicted by using the substrate plane to incise the {111} crystallographic planes.

**Figure 6:**
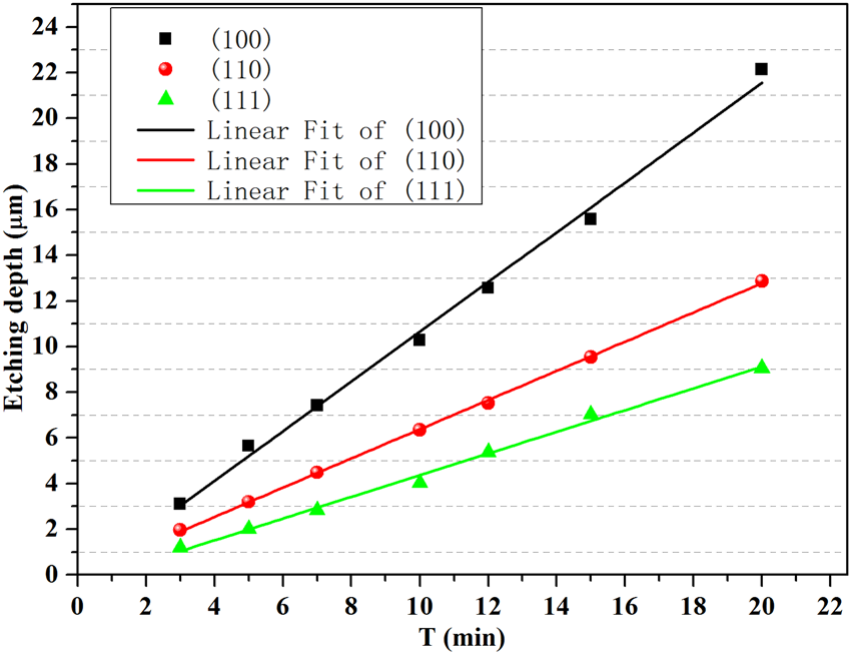
The etching depth of the three oriented wafers versus the etching time and the corresponding fitted curves by Cu based acid solutions.

In order to thoroughly understand the anisotropic etching mechanism of CACE, the etching rate of the three oriented wafers is characterized. The average etching rate (${R}_{{\rm{av}}}$) was calculated as:^30^

$${R}_{{\rm{av}}}=\frac{{\rm{\Delta }}m}{2{\rho }_{{\rm{Si}}}St}$$

where ${\rm{\Delta }}m$ is the mass loss of the sample, ${\rho }_{{\rm{Si}}}$ is the mass density of crystalline Si, *S* is the Si surface area, and *t* is the etching time. Figure 6 shows the etching depth of the three oriented wafers versus the etching time and the corresponding fitted curves by Cu based acid solutions. The etching depth varies linearly with the etching time, in other words, the ${R}_{{\rm{av}}}$ basically remains unchanged with the prolongation of etching time. The ${R}_{{\rm{av}}}$ of Si (100), (110), (111) oriented wafers is 1.09 μm/min, 0.64 μm/min, 0.54 μm/min, respectively. The surface bond density of Si (100), (110) and (111) is 1.36, 0.96 and 0.78 × 10^15^/cm^2^^31^. Higher surface bond density leading to higher probability of Cu^2+^ ions to capture electrons, which makes faster etching rate. The ${R}_{{\rm{av}}(100)}$/${R}_{{\rm{av}}(111)}$ ratio known as anisotropy factor is 2.01 which is very close to the value (1.74) as that of the ratio between the free bond density of Si (100) and (111) planes. While, for certain alkaline etchants, the etch rate of the [111] direction is effectively zero. Thus, the anisotropic factor is usually over 100 for alkaline etchants. The etching rate of (111) substrate by Cu assisted anisotropic etching is much faster than alkali etching, which may have many potential applications. According to Figs 3 to 5, more Cu nanoparticles are deposited on (100) than (110) and (111) surface, thus more reacting points are formed on (100) substrate leading to the faster etching of (100) plane.

**Figure 7:**
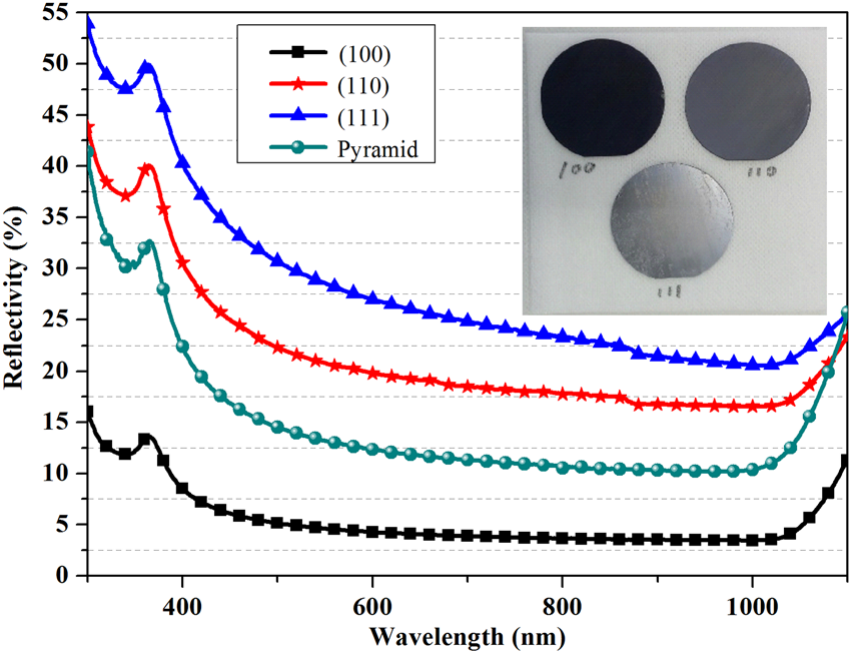
Reflectance spectra of the three structure obtained via Cu assisted anisotropic etching for 15 min on Si (100), (110) and (111) substrate and the upright pyramids obtained by alkaline etchant. The inset shows photograph of the three samples.

The reflectance spectra of the three structures obtained via Cu assisted anisotropic etching for 15 min on Si (100), (110) and (111) substrate and the upright pyramids obtained by alkaline etchant are shown in Fig. 7. The average reflectance ($\bar{R}$) of inverted pyramid obtained on Si (100) substrates is lower than 5%, while the $\bar{R}$ of upright pyramid is 12%. The superior light-trapping characteristic is original from the inverted pyramidal structure that about 37% of the incoming light undergoes a triple bounce before being reflected away^32^. While, the $\bar{R}$ of Si (110) and (111) substrates were larger than 20%, due to the poor light management effect. As can been seen from the photos, the appearance of Si (100) substrate is black and the appearance of Si (110) substrate is gray, while the appearance of Si (111) substrate is a bit bright because most of the incoming light was reflected away after the first bounce. By thorough understanding the etching mechanism of CACE, the morphology, size and surface roughness of the etched inverted pyramids are under control. The low-cost micron-sized inverted pyramid texture technique results in surfaces with excellent optical and electronic properties^33^, and is therefore well suited to high-efficiency silicon solar cells.

In summary, the different behaviors of anisotropic etching of Si by alkaline and Cu based acid etchant have been systematically investigated. Thanks to the lower reduction potential of Cu^2+^/Cu and different electronic properties of different Si planes, the deposition of Cu nanoparticles shows orientation-dependent, which leads to the anisotropic etching of Si substrate. Besides, both anisotropic etching and localized etching process contribute to the formation of inverted pyramid. The obtained structure is bounded by the Si {111} planes, no matter what orientation of Si substrate is. Different from alkali etching, the value of anisotropic factor of CACE is almost the same value as that of the ratio between the free bond density of Si (100) and (111) planes. Much faster etching rate (0.54 μm/min) on Si (111) plane has been realized by Cu based acid solutions compared to almost zero etching rate by alkaline solutions. What’s more, the superior structural characteristic fabricated on Si (100) surface has huge application in photovoltaic cell mass production.

## Methods

Boron-doped (1–3 Ω·cm), 500 μm thick, (100), (110) and (111) oriented, double polished Si wafers were thoroughly rinsed in acetone to remove any organic contaminants and then rinsed with deionized water before etching. The upright pyramid texture was obtained by etching in alkaline solutions containing 2 wt% potassium hydroxide (KOH) and 10 vol% IPA. Meanwhile, we got the inverted pyramid texture by using Cu based acid solutions containing 5 mM Cu(NO_3_)_2_, 4.6 M HF, and 0.55 M H_2_O_2_ at 50 °C. The residual Cu nanoparticles were removed away using concentrated nitric acid in a sonication bath. The morphologies and structures of the wafers were characterized by a Hitachi S-4800 scanning electron microscope (SEM). The hemispheric total reflectance for normal incidence in wavelength from 300 nm to 1000 nm was measured using a Varian Cary 5000 spectrophotometer with an integrating sphere.
